# Evaluation of systemic inflammatory response following transcatheter aortic valve replacement: a pathway to rational antibiotic use

**DOI:** 10.1007/s15010-025-02485-0

**Published:** 2025-02-07

**Authors:** Henning Guthoff, Valerie Lohner, Ute Mons, Julia Götz, Hendrik Wienemann, Jan Wrobel, Stephan Nienaber, Sascha Macherey-Meyer, Philipp von Stein, Stephan Baldus, Matti Adam, Maria Isabel Körber, Norma Jung, Victor Mauri

**Affiliations:** 1 Heart Center, Department III of Internal Medicine, Faculty of Medicine and University Hospital Cologne, University of Cologne, Kerpener Str. 62, 50937 Cologne, Germany; 2 Cardiovascular Epidemiology of Aging, Department III of Internal Medicine, Faculty of Medicine and University Hospital Cologne, University of Cologne, Cologne, Germany; 3 Division of Infectious Diseases, Department I of Internal Medicine, Faculty of Medicine and University Hospital Cologne, University of Cologne, Cologne, Germany

**Keywords:** Transcatheter aortic valve replacement, TAVR, TAVI, Inflammation, Infection, Antibiotic stewardship

## Abstract

**Purpose:**

Elevations in inflammatory markers after transcatheter aortic valve replacement (TAVR) often lead to preemptive antibiotic therapy (ABT). Distinguishing between physiological inflammatory reaction and true infection is crucial for rational ABT use.

**Methods:**

This retrospective study included 1275 consecutive TAVR patients from January 2020 to July 2022. Infectious foci, ABT administration, and inflammatory markers over seven days post-procedure were evaluated. Using multivariable logistic regression, predictors for infection were identified and integrated into the Risk of Infection After TAVR (RIAT) score.

**Results:**

An infectious focus was retrospectively identified in 2.6% of patients, while 11.4% received ABT. Distinct trends in body temperature (BT), white blood cells (WBC), and C-reactive protein (CRP) were noted, with BT and WBC peaking on day 1 and CRP on day 3. Significant predictors of infection included a rise in BT of ≥ 0.2 °C between day 1 and 3 (odds ratio [OR] 3.08, 95% confidence interval [CI] 1.38–6.88, *p* = 0.006), elevated WBC counts ≥ 12 × 10^9^/L (OR 3.77, 95% CI 1.67–8.48, *p* = 0.001), and CRP levels ≥ 80 mg/L (OR 5.72, 95% CI 2.59–12.64, *p* < 0.001) within three days after TAVR. Integrating these into the RIAT score revealed an infection probability of 1.5% for scores 0–3 points, 9.2% for scores 4–6 points, and 54.5% for scores 7–8 points.

**Conclusion:**

Our findings indicate significant ABT overuse among TAVR recipients, likely due to misinterpretation of postoperative physiological reactions. Incorporating specific changes and thresholds of BT, WBC, and CRP post-TAVR into the RIAT score improved risk prediction for infection, underscoring its utility in enhancing antibiotic stewardship in this growing patient population.

**Supplementary Information:**

The online version contains supplementary material available at 10.1007/s15010-025-02485-0.

## Introduction

Transcatheter aortic valve replacement (TAVR) has emerged as a leading technique for the treatment of aortic stenosis [1, 2]. Despite its minimally invasive nature, TAVR procedures are associated with a rise in body temperature (BT), elevated white blood cell (WBC) counts, and increased C-reactive protein (CRP) levels. Although these elevations may often reflect a physiological postoperative inflammatory reaction rather than an infection, they frequently prompt the initiation of antibiotic therapy (ABT) to prevent infections associated with the newly implanted prosthesis [3–6]. Notably, while the incidence of endocarditis post-TAVR is low at approximately 0.9% annually, about 15.7% of endocarditis cases occur within the first 30 days after the procedure [7, 8]. Additionally, TAVR endocarditis is associated with a significant mortality rate of almost 50% within a year, emphasizing the importance of its prevention [7]. The excessive use of ABT, however, poses risks including adverse reactions and antibiotic resistance, with the latter being declared as one of the top ten global health threats by the World Health Organization [9]. Therefore, the discrimination of physiological postoperative changes from true infections is essential [10, 11]. 

Recent studies indicate that the rate of in-hospital infections following TAVR ranges from 11 to 20% [4, 12–15]. This contrasts with the general incidence of in-hospital infections in Western countries, which are reported to be around 4%, and with the incidence of in-hospital infections after cardiac surgery, which are around 4.2% [16, 17]. These substantially higher incidence rates after TAVR might be attributable, at least in part, to the noticeable lack of detailed data on typical post-TAVR physiological reactions, which could mimic symptoms of infectious complications. The prevalence of aortic valve disease and the increasing adoption of TAVR as a routine intervention necessitate a better understanding of these postoperative changes [18–20]. 

The present investigation aims to enhance our understanding of the physiological response to TAVR and to refine the differentiation between postoperative inflammatory and infectious states, ultimately enabling better antibiotic stewardship and patient care post-TAVR.

## Methods

### Study population

This study included 1275 consecutive patients receiving TAVR for severe native aortic stenosis at a high-volume center in Germany from January 2020 to July 2022. Valve-in-valve procedures (40 patients) and patients on ABT before the procedure (12 patients) were excluded. Only infections or ABT initiation within seven days post-TAVR were considered and systematically analyzed.

The decision to perform TAVR was made by the institutional heart team. Procedural specifics (e.g., access route, balloon aortic valvuloplasty, postdilation) were at the operators’ discretion. All patients received periprocedural antibiotic prophylaxis with a first- or second-generation cephalosporin, or Vancomycin/Clindamycin in case of allergies. Procedures used monoplane fluoroscopy, typically under conscious sedation and local anesthesia. For the procedure, urinary catheters, peripheral and central venous accesses, a peripheral arterial sheath, and a temporary pacemaker (inserted via a central venous catheter) were used. The temporary pacemaker was removed immediately post-procedure unless otherwise indicated. After procedure, patients were monitored for at least 24 h in an intensive care unit (ICU), with all invasive lines removed within this period unless clinically indicated otherwise.

Retrospective data collection was approved by the institutional review board (19-1032_2) and was conducted in accordance with the Declaration of Helsinki.

### Data collection

Clinical and procedural data were collected retrospectively from medical records. Inflammatory parameters were assessed pre-procedurally and for seven days after the procedure. Fever was defined as BT ≥ 38 °C. The initiation of ABT, specific substances used, and therapy duration were recorded. Clinical documentation, including infectious symptoms, microbiological, and radiological findings, was systematically collected for each patient who received ABT.

Major procedural complications were defined as a composite of new pacemaker implantation, stroke/transient ischemic attack (TIA), ≥type 2 bleeding, or major vascular complications as defined by the Valve Academic Research Consortium 3 (VARC3) [21]. 

### Assessment of antibiotic therapy and infectious foci

Patients who received ABT were systematically evaluated by infectious disease specialists to confirm or exclude an infectious focus. This included clinical presentations (as documented in the medical record), laboratory tests, microbiological, and imaging studies. Definitions and diagnostic criteria for infectious foci are provided in Supplemental Table S1. All patients receiving ABT post-TAVR (ABT_a_) were classified into those with a retrospectively confirmed infection (ABT_cf._) and those without (ABT_nf_) for further analyses.

### Statistical analysis

Categorical variables were presented as percentages, and continuous variables as means with standard deviations (SD) or medians with interquartile ranges (IQR), as appropriate. Categorical variables were compared using chi-square tests. For continuous variables, unpaired Student’s t-tests were used for normally distributed variables, and Mann-Whitney U tests for asymmetrically distributed variables. Normality was determined using the Kolmogorov-Smirnov test.

To compare the trends of BT, WBC, and CRP across different time points and between groups, a two-way ANOVA was employed. This yielded coefficients for time, group classification, and time by group interaction. Significant group differences indicated by ANOVA were followed by unpaired, two-tailed t-tests for specific time points.

Predictors of an infectious focus were examined using binary logistic regression of clinically relevant variables. Variables with *p* < 0.1 in the univariable model were included in a multivariable model. Multicollinearity was assessed prior to the regression analysis and found to be absent. Inflammatory parameters within the first three days post-TAVR were specifically included to ensure accurate identification of infections in the early phase post-TAVR, during which the majority of ABT were initiated. Cutoff values for WBC and CRP were set at 12 × 10^9^/L and 80 mg/L, respectively, as these thresholds approximately correspond to the uppermost quintiles. Increase in BT was defined as a rise of ≥ 0.2 °C to account for common standard deviations of measurement instruments. Change in BT was analyzed from day 1 to 3, as the course after the peak value, typically occurring on day 1, was of particular interest. Odds ratios (OR) and 95% confidence intervals (CI) are reported.

For the development of the Risk of Infection After TAVR (RIAT) score, the cohort was randomly divided using stratified sampling into a training (70%) and a validation cohort (30%), while maintaining an even distribution of confirmed infections (training cohort: 23/879 = 2.61%; validation cohort: 10/396 = 2.52%). Logistic regression-identified factors were incorporated into the score model with varying manifestations and point values. Receiver operating characteristic (ROC) analysis and area under the curve calculations (AUC) assessed the score’s effectiveness, with an AUC > 0.8 deemed satisfactory [22]. 

Statistical analyses were performed with SPSS version 28.0 (IBM, Amonk, USA), GraphPad Prism version 9.5 (GraphPad Software, San Diego, USA) and R version 4.3.0 (R Core Team, Vienna, Austria, 2023). A p-value < 0.05 was considered statistically significant.

## Results

### Patient characteristics

**Fig. 1:**
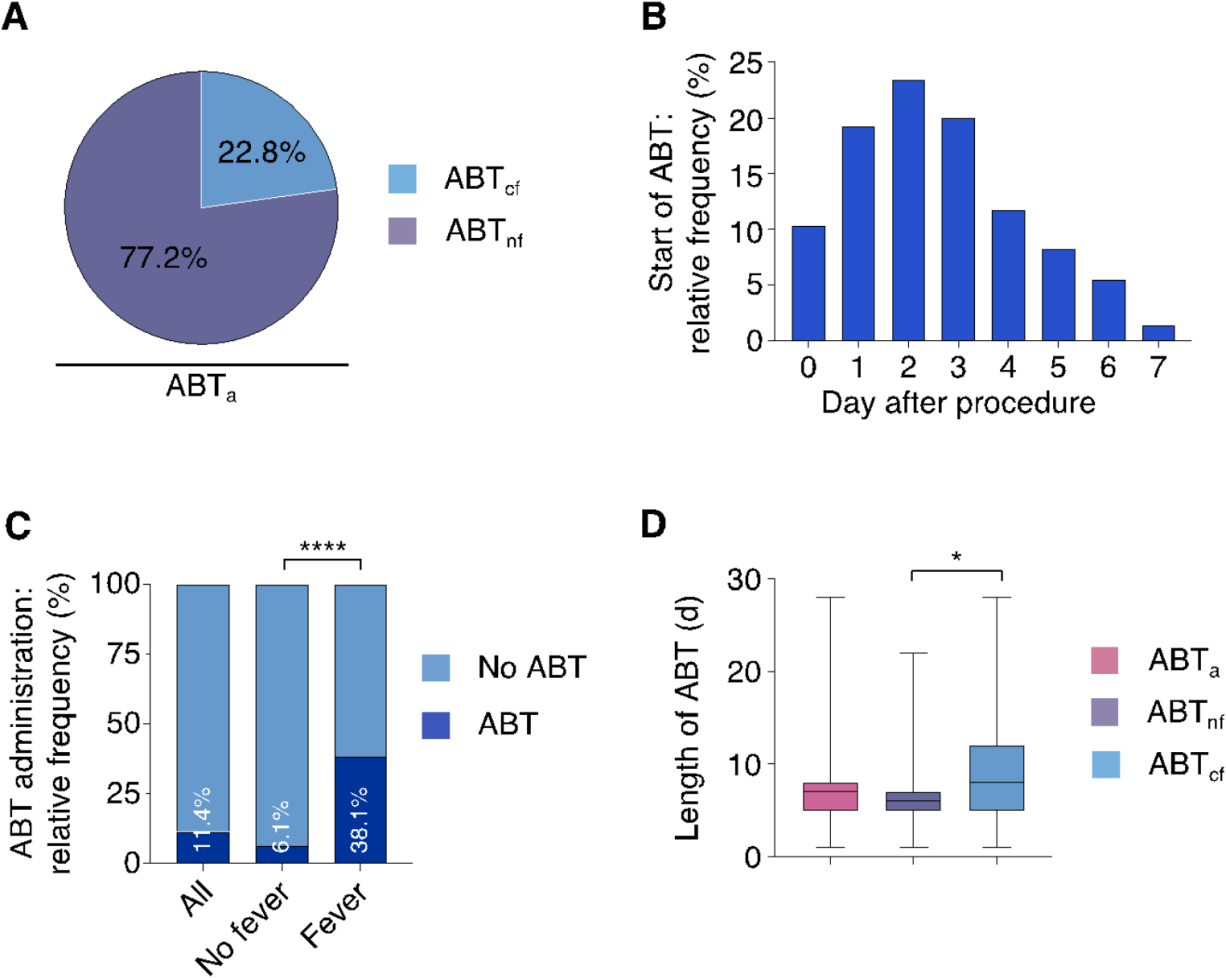
(**A**) Proportion of patients with and without a confirmed infectious focus among those receiving ABT. (**B**) Relative frequency of ABT initiation on the indicated post-procedural days. (**C**) Relative frequency of ABT administration in the overall patient cohort, and stratified by the presence and absence of fever. (**D**) Duration of ABT in days in all patients who received ABT and in those with and without a confirmed infectious focus. Data are shown as median (central line within each box), interquartile range (box), and range (whiskers). *p < 0.05, ****p < 0.0001. Abbreviations: ABT– Antibiotic Therapy, ABT_a_– all patients receiving ABT, ABT_cf_– patients receiving ABT with a confirmed focus, ABT_nf_– patients receiving ABT without a confirmed focus, BT– Body Temperature, CRP– C-Reactive Protein, WBC– White Blood Cells

Patients showed typical characteristics of a contemporary TAVR cohort: 569 were female (44.6%) with a mean age of 81.5 ± 6.1 years and a mean STS score of 4.3 ± 4.2%. Further baseline characteristics are detailed in Table 1. Within seven days post-TAVR, 145 patients (11.4%) received ABT (ABT_a_ group). Of these individuals, retrospective analysis identified a confirmed infectious focus in 33 (22.8% = ABT_cf._ group), while 112 patients received ABT without a retrospectively confirmed infectious focus (77.2% = ABT_nf_ group) (Fig. 1A).

**Table 1:** Baseline characteristics of the entire study population and stratified by ABT administration

Parameter	All	ABT		*P*
		No	Yes (ABT_a_)	
*N*	1275	1130	145	
Age - years	81.5 ± 6.1	81.6 ± 6.0	80.8 ± 6.8	0.419
Females	569 (44.6%)	506 (44.8%)	63 (43.5%)	0.791
BMI - kg/m^2^	27.0 ± 5.1	27.0 ± 5.0	27.4 ± 5.8	0.533
Average BT over 7d post-TAVR - °C	36.7 ± 0.4	36.7 ± 0.4	37.1 ± 0.5	**< 0.001**
Maximum BT within 7d post-TAVR - °C	37.4 ± 0.6	37.3 ± 0.5	38.0 ± 0.7	**< 0.001**
Average WBC over 7d post-TAVR - x10^9^/L	8.7 ± 4.2	8.4 ± 3.0	11.1 ± 8.7	**< 0.001**
Maximum WBC within 7d post-TAVR - x10^9^/L	10.7 ± 5.8	10.1 ± 4.1	15.1 ± 12.0	**< 0.001**
Average CRP level over 7d post-TAVR - mg/L	30.5 ± 30.3	24.9 ± 22.5	73.9 ± 44.4	**< 0.001**
Maximum CRP level within 7d post-TAVR - mg/L	55.2 ± 53.8	44.8 ± 39.0	136.1 ± 78.5	**< 0.001**
Fever post-TAVR (BT ≥ 38 °C)	210 (16.7%)	130 (11.5%)	80 (55.2%)	**< 0.001**
GFR - ml/min	59 ± 21	59 ± 21	57 ± 22	0.357
NYHA FC				**0.013**
I	38 (3.1%)	35 (3.2%)	3 (2.1%)	
II	331 (27.0%)	307 (28.4%)	24 (16.8%)	
III	776 (63.3%)	668 (61.7%)	108 (75.5%)	
IV	80 (6.5%)	72 (6.7%)	8 (5.6%)	
EuroSCORE II - %	4.3 ± 3.9	4.2 ± 3.9	4.7 ± 4.2	0.072
STS score - %	4.3 ± 4.2	4.3 ± 4.3	4.5 ± 3.2	0.054
Comorbidities				
Hypertension	1106 (87.2%)	977 (86.9%)	129 (89.0%)	0.589
Diabetes mellitus	353 (27.8%)	313 (27.9%)	40 (27.6%)	0.947
Peripheral arterial disease	223 (17.6%)	193 (17.2%)	30 (20.7%)	0.297
Coronary heart disease	765 (60.3%)	683 (60.8%)	82 (56.6%)	0.367
Atrial fibrillation	505 (39.8%)	432 (38.4%)	73 (50.3%)	**0.007**
COPD	139 (11.0%)	114 (10.1%)	25 (17.2%)	**0.015**
Median length of hospital stay - days	7 (5–10)	7 (5–9)	12 (8–19)	**< 0.001**
Median length of ICU stay - days	2 (1–3)	2 (1–2)	3 (1–6)	**< 0.001**
Self-expanding device	840 (65.9%)	751 (66.5%)	89 (61.4%)	0.228
Major complication	266 (20.9%)	200 (17.7%)	66 (45.5%)	**< 0.001**

Major complications include new pacemaker implantation, stroke/TIA, and events classified as ≥ type 2 bleeding or major vascular complications as defined by VARC3

Abbreviations: ABT– Antibiotic Therapy, BMI– Body Mass Index, BT– Body Temperature, COPD– Chronic Obstructive Pulmonary Disease, CRP– C-Reactive Protein, GFR– Glomerular Filtration Rate (according to Chronic Kidney Disease Epidemiology Collaboration), ICU– Intensive Care Unit, NYHA FC– New York Heart Association Functional Class, TIA– Transient Ischemic Attack, VARC3– Valve Academic Research Consortium-3, WBC– White Blood Cell Count

The ABT_a_ group had higher maximum BT, WBC counts, and CRP levels, as well as longer hospital and ICU stays than those without ABT. Higher rates of atrial fibrillation, chronic obstructive pulmonary disease (COPD), as well as more severe New York Heart Association functional class (NYHA FC), were observed. Major procedural complications were more frequent in the ABT_a_ group (Table 1).

In the ABT_cf._ group, higher maximum BT, WBC counts, and CRP levels were observed compared to the ABT_nf_ group. They also had longer hospital and ICU stays. Although major procedural complications were numerically more frequent in the ABT_cf._ group, this difference was not statistically significant. Other baseline characteristics such as age, sex, and comorbidities did not differ significantly between groups (Table 2). Within the ABT_cf._ group, 19 patients met sepsis criteria, and 8 progressed to septic shock. Of these, 3 patients died (Supplemental Figure S1).

**Table 2:** Baseline characteristics of the entire study population and stratified by the presence or absence of a confirmed infectious focus

Parameter	All	All		*P*
		No focus	Confirmed focus (ABT_cf._)	
*N*	1275	1242	33	
*Age - years*	81.5 ± 6.1	81.6 ± 6.0	79.6 ± 7.5	0.219
*Females*	569 (44.6%)	558 (44.9%)	11 (33.3%)	0.216
*BMI - kg/m* ^*2*^	27.0 ± 5.1	27.0 ± 5.1	29.1 ± 6.5	0.089
*Average BT * *over 7d post-TAVR - °C*	36.7 ± 0.4	36.7 ± 0.4	37.1 ± 0.5	**< 0.001**
*Maximum BT* *within 7d post-TAVR - °C*	37.4 ± 0.6	37.4 ± 0.6	38.0 ± 0.7	**< 0.001**
*Average WBC* *over 7d post-TAVR - x10* ^*9*^ */L*	8.7 ± 4.2	8.7 ± 4.2	11.1 ± 3.7	**< 0.001**
*Maximum WBC* *within 7d post-TAVR - x10* ^*9*^ */L*	10.7 ± 5.8	10.5 ± 5.7	15.8 ± 6.2	**< 0.001**
*Average CRP level* *over 7d post-TAVR - mg/L*	30.5 ± 30.3	29.0 ± 27.9	87.0 ± 53.6	**< 0.001**
*Maximum CRP level* *within 7d post-TAVR - mg/L*	55.2 ± 53.8	52.4 ± 49.3	162.4 ± 92.3	**< 0.001**
*Fever post-TAVR (BT ≥ 38 °C)*	210 (16.7%)	196 (15.8%)	14 (42.4%)	**< 0.001**
*GFR - ml/min*	59 ± 21	59 ± 21	56 ± 24	0.497
*NYHA FC*				0.296
*I*	38 (3.1%)	37 (3.1%)	1 (3.2%)	
*II*	331 (27.0%)	327 (27.4%)	4 (12.1%)	
*III*	776 (63.3%)	751 (63.0%)	24 (72.7%)	
*IV*	80 (6.5%)	78 (6.5%)	2 (6.1%)	
*EuroSCORE II - %*	4.3 ± 3.9	4.3 ± 3.9	4.4 ± 4.5	0.917
*STS score - %*	4.3 ± 4.2	4.3 ± 4.2	4.3 ± 3.3	0.663
*Comorbidities*				
*Hypertension*	1106 (87.2%)	1076 (87.1%)	29 (87.9%)	0.791
*Diabetes mellitus*	353 (27.8%)	340 (27.5%)	12 (36.4%)	0.166
*Peripheral arterial disease*	223 (17.6%)	218 (17.6%)	5 (15.2%)	0.988
*Coronary heart disease*	765 (60.3%)	749 (60.6%)	15 (45.5%)	0.206
*Atrial fibrillation*	505 (39.8%)	490 (39.6%)	15 (45.5%)	0.589
*COPD*	139 (11.0%)	136 (11.0%)	3 (9.1%)	0.959
*Median length of hospital stay * *- days*	7 (5–10)	7 (5–9)	13 (9–21)	**< 0.001**
*Median length of ICU stay * *- days*	2 (1–3)	2 (1–3)	3 (1–8)	**< 0.001**
*Self-expanding device*	840 (65.9%)	818 (65.9%)	21 (63.6%)	1.000
*Major complication*	266 (20.9%)	255 (20.5%)	11 (33.3%)	0.083

Major complications include new pacemaker implantation, stroke/TIA, and events classified as ≥ type 2 bleeding or major vascular complications as defined by VARC3

Abbreviations: BMI– Body Mass Index, BT– Body Temperature, COPD– Chronic Obstructive Pulmonary Disease, CRP– C-Reactive Protein, GFR– Glomerular Filtration Rate (according to Chronic Kidney Disease Epidemiology Collaboration), ICU– Intensive Care Unit, NYHA FC– New York Heart Association Functional Class, TIA– Transient Ischemic Attack, VARC3– Valve Academic Research Consortium-3, WBC– White Blood Cell Count

### Analysis of ABT administration and confirmation of infectious foci

The majority of ABT was initiated on day 2 post-TAVR, accounting for 24.4% of patients (Fig. 1B). Piperacillin/Tazobactam was the most frequently used antibiotic, followed by Ceftriaxone and Ampicillin/Sulbactam (Supplemental Table S2). Initiation of ABT was more common in patients with fever than without (38.1% vs. 6.1%, *p* < 0.001) (Fig. 1C). Median ABT duration was 7 days (IQR: 5–8), and longer in patients with a confirmed focus (8 days, IQR: 5–12) than without (6 days, IQR: 5–7, *p* = 0.017) (Fig. 1D). Among confirmed infections, urinary tract infections (14 cases) and pneumonia (10 cases) were most common. Supplemental Table S3 lists initial diagnoses and retrospectively confirmed infectious diagnoses. Supplemental Figure S2 summarizes the proportion of appropriate diagnostic testing performed for each suspected diagnosis. A list of the pathogens identified in BC can be found in Supplemental Table S4.

### Inflammatory reactions post-TAVR

**Fig. 2:**
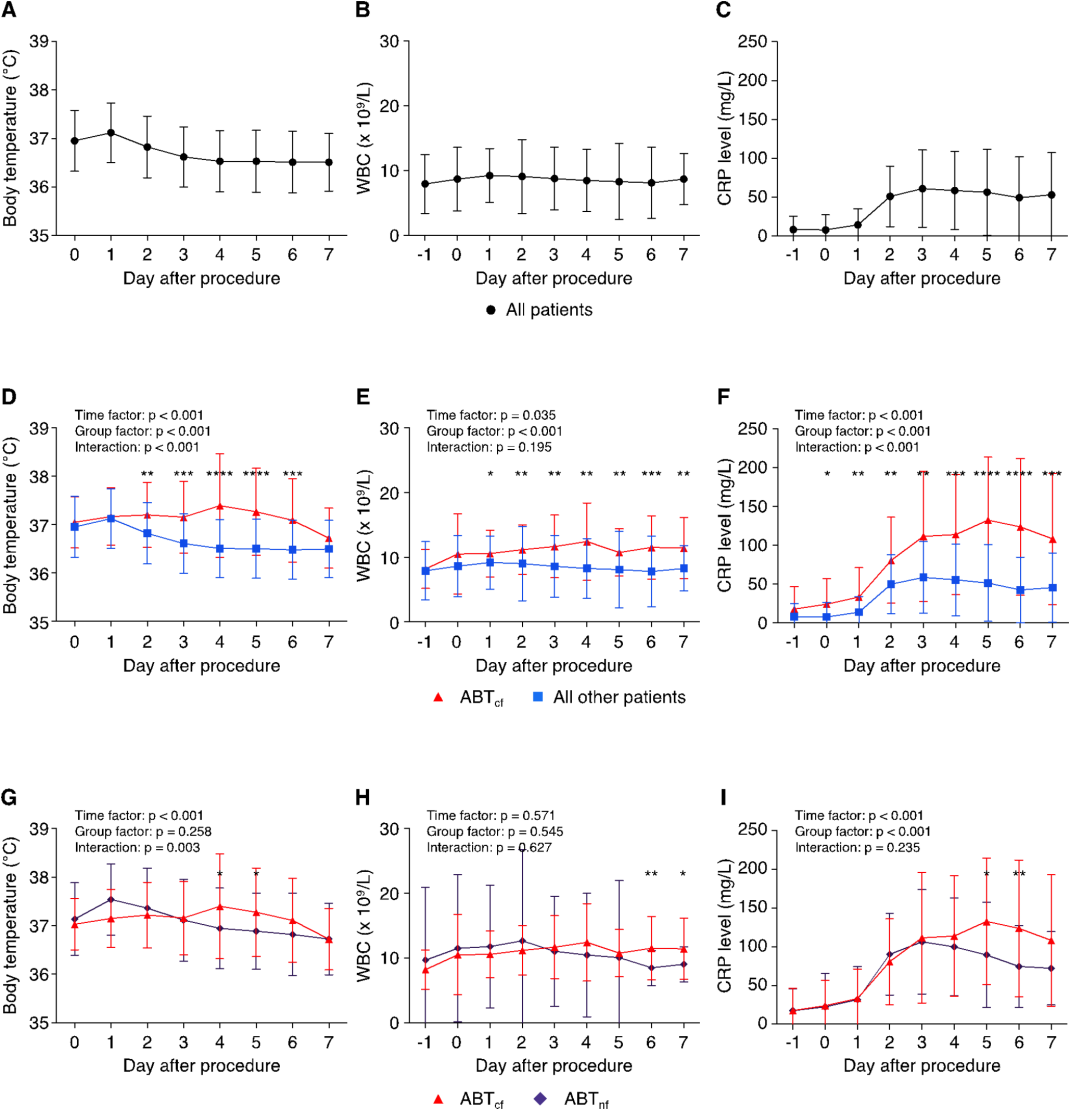
(**A**-**C**) Trajectories of BT, WBC, and CRP levels in the overall patient cohort. (**D**-**F**) Trajectories stratified by the presence (ABT_cf._) and absence of a confirmed infectious focus. (**G**-**I**) Respective trends according to the presence of an infectious focus (ABT_cf._) and administration of ABT without a confirmed focus (ABT_nf_). Time factor assesses variations in parameters over time while group factor focuses on differences between groups. Interaction analysis determines whether the effect of time on parameters varies between groups. Unpaired, two-tailed t tests to compare specific time points between groups were performed, if ANOVA indicated significant differences between groups. *p < 0.05, **p < 0.01, ***p < 0.001, ****P < 0.0001. Abbreviations: ABT– Antibiotic Therapy, ABT_a_– all patients receiving ABT, ABT_cf._– patients receiving ABT with a confirmed focus, ABT_nf_– patients receiving ABT without a confirmed focus, d– days

Following TAVR, distinct trends in BT, WBC, and CRP levels were observed in the overall cohort. BT and WBC peaked on day 1 (37.1 ± 0.6 °C and 9.3 ± 4.2 × 10^9^/L), while CRP peaked on day 3 (60.9 ± 49.7 mg/L) (Fig. 2A-C).

In the ABT_cf._ group, compared to all other patients, BT, WBC, and CRP levels were significantly higher with delayed peaks (two-way ANOVA group factor: *p* < 0.05, respectively). BT peaked on day 4 (37.4 ± 1.1 °C), WBC counts on day 4 (12.4 ± 6.0 × 10^9^/L), and CRP levels on day 5 (132.7 ± 81.3 mg/L) (Fig. 2D-F).

In the ABT_cf._ and ABT_nf_ groups, BT, WBC, and CRP were similar for the first three days. From day 3, trends separated visually. Therefore, time courses from days 0 to 3 and 3 to 7 were compared individually. No significant differences were observed in the first three days for any parameter (BT: *p* = 0.057; WBC: *p* = 0.463; CRP: *p* = 0.950, group factor, respectively). From day 3 to 7, significant differences emerged for BT (group factor: *p* = 0.003), WBC (group factor: *p* = 0.023), and CRP (group factor: *p* < 0.001), with all three parameters showing distinct trajectories between the two groups and delayed peaks in the ABT_cf._ patients (Fig. 2G-I).

### Predictors of infection

In a multivariable binary logistic regression analysis, including clinically relevant variables with *p* < 0.1 in the univariable model, a rise in BT of ≥ 0.2 °C between day 1 and 3, elevated WBC counts ≥ 12 × 10^9^/L, and CRP levels ≥ 80 mg/L within three days following TAVR emerged as independent predictors of infection (Table 3).

**Table 3:** Uni- and multivariable binary logistic regression analysis of factors predictive of a confirmed infectious focus post-TAVR

Parameter	OR	95% CI	*P*	OR	95% CI	*P*
	*Univariable model*			*Multivariable model*		
*Age*	0.95	0.91-1.00	**0.065**	0.97	0.92–1.02	0.230
*Female Sex*	0.61	0.30–1.28	0.190			
*BMI*	1.07	1.01–1.12	**0.020**	1.02	0.96–1.08	0.575
*GFR (CKD EPI)*	1.00	0.98–1.01	0.513			
*NYHA FC ≥ III*	2.37	0.91–6.21	**0.079**	1.93	0.71–5.23	0.196
*Hypertension*	1.49	0.45–4.93	0.516			
*Diabetes mellitus*	1.71	0.84–3.48	0.137			
*Peripheral artery disease*	1.20	0.49–3.14	0.712			
*Coronary artery disease*	1.63	0.82–3.27	0.164			
*Atrial fibrillation*	1.27	0.63–2.54	0.502			
*COPD*	1.24	0.37–4.11	0.729			
*Major complication*	1.94	0.93–4.04	**0.079**	0.89	0.39–2.03	0.782
*BT rise d1-3 ≥ 0.2 °C*	3.31	1.58–6.95	**0.002**	3.08	1.38–6.88	**0.006**
*WBC ≥ 12 × 10* ^*9*^ */L within 3d*	7.03	3.37–14.68	**< 0.001**	3.77	1.67–8.48	**0.001**
*CRP ≥ 80 mg/L within 3d*	9.57	4.63–19.78	**< 0.001**	5.72	2.59–12.64	**< 0.001**

All variables with a p-value < 0.1 in the univariable model were included in the subsequent multivariable analysis. Major complications include new pacemaker implantation, stroke/TIA, and events classified as ≥ type 2 bleeding or major vascular complications as defined by VARC3

Abbreviations: BMI– Body Mass Index, COPD– Chronic Obstructive Pulmonary Disease, CRP– C-Reactive Protein, GFR– Glomerular Filtration Rate (according to Chronic Kidney Disease Epidemiology Collaboration (CKD EPI)), NYHA FC– New York Heart Association Functional Class, TIA– Transient Ischemic Attack, VARC3– Valve Academic Research Consortium-3, WBC– White Blood Cell Count

### Development of the risk of infection after TAVR (RIAT) score

Given the similar trajectories of BT, WBC and CRP within the first three days post-TAVR between patients with pronounced inflammatory reactions (ABT_nf_) and those with confirmed infections (ABT_cf._) (Fig. 2G-I), we aimed to integrate these factors into a risk stratification model. Therefore, the patient cohort was randomly divided into a training and validation cohort (Supplemental Table S5).

**Fig. 3:**
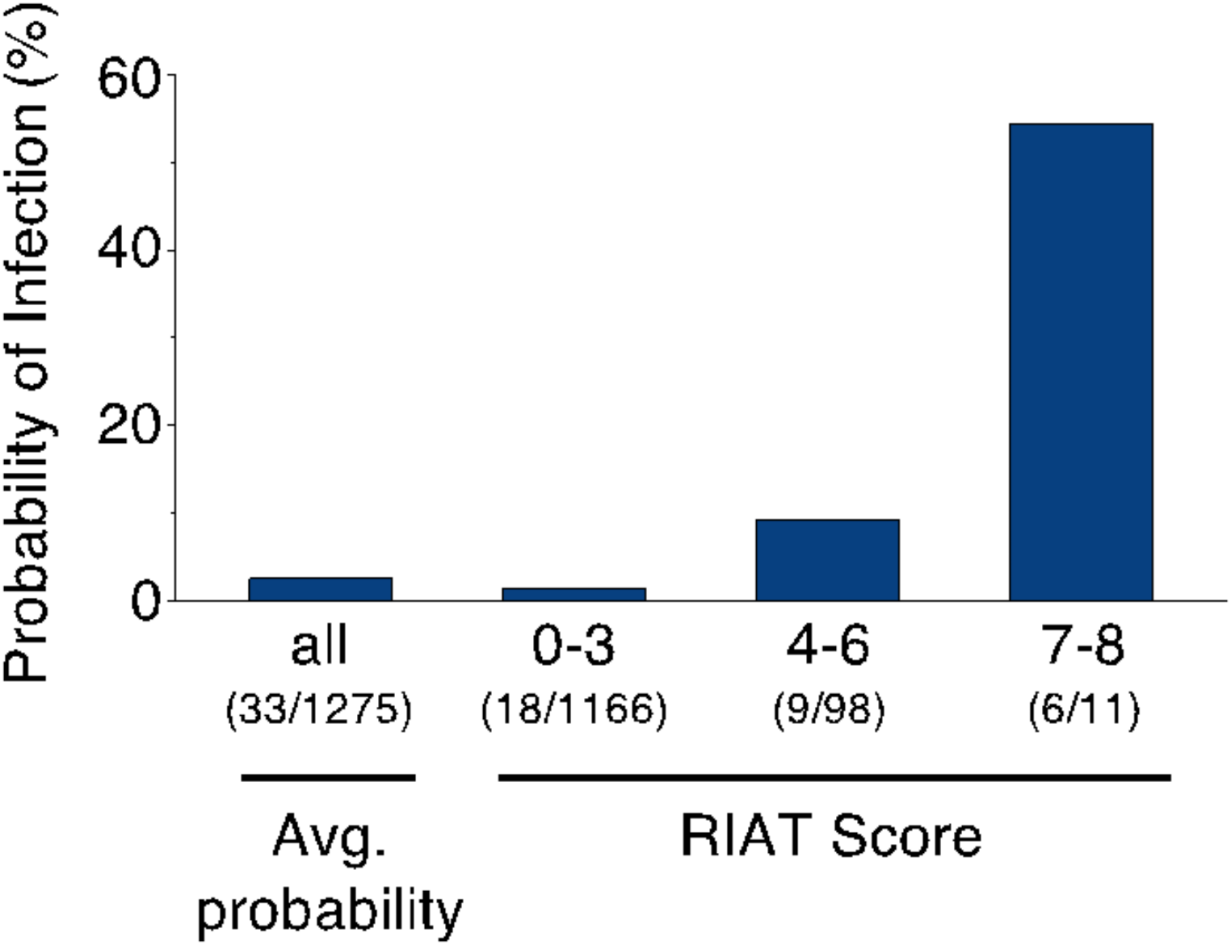
Probability of infection across the overall patient cohort and stratified by points in the RIAT Score. Abbreviations: Avg– average, RIAT– Risk of Infection After TAVR

Changes in BT from day 1 to 3, peak WBC counts, and CRP levels within the first three days were combined to create the RIAT score (Table 4). ROC analyses showed an AUC of 0.833 (95% CI 0.737–0.929) in the training cohort and 0.801 (95% CI 0.654–0.948) in the validation cohort. Across the entire cohort, the AUC was 0.823 (95% CI 0.743–0.904) (Supplemental Figure S3). The RIAT score demonstrated a reliable gradient of infection probability with increasing scores in the total cohort: 1.5% for scores 0–3, 9.2% for scores 4–6, and 54.5% for scores 7–8 (Fig. 3). Additionally, the sensitivity, specificity, and positive and negative predictive values for the corresponding cutoffs ≥ 4 and ≥ 7 are detailed in Supplemental Table S6.

**Table 4:** Parameters of the risk of infection after TAVR (RIAT) score with the subsequent point values

Clinical parameter	Criteria	Score
*BT change d1-3 post-TAVR*	Decline ≥ 0.2 °C	**0**
	No change (± 0.1 °C)	**1**
	Increase ≥ 0.2 °C	**2**
*Max WBC within 3d post-TAVR*	≤ 12 × 10^9^/L	**0**
	12–16 × 10^9^/L	**1**
	> 16 × 10^9^/L	**3**
*Max CRP within 3d post-TAVR*	≤ 80 mg/L	**0**
	80–120 mg/L	**1**
	> 120 mg/L	**3**

Abbreviations: BT: Body Temperature, CRP– C-Reactive Protein, WBC– White Blood Cell Count

## Discussion

TAVR recipients with endocarditis have a poor prognosis, with mortality rates of up to 50% within a year [7]. However, growing antibiotic resistance resulting from undifferentiated ABT use poses one of the greatest risks to global health today [9]. In this study, we analyzed the incidence of early post-interventional infections within seven days post-TAVR following strict diagnostic criteria. We then contrasted these findings with the initial clinical assessment and the frequency of ABT administration, revealing substantial antibiotic overuse. To the best of our knowledge, this investigation is the largest and most detailed analysis to date of the physiological course of key inflammation indicators post-TAVR, including BT, WBC, and CRP. It elucidates their roles and limitations in distinguishing between normal postoperative responses and genuine infections. Importantly, by incorporating these indicators into the RIAT score, our study proposes a simple risk assessment tool to more precisely estimate the probability of early infections after TAVR.

The main findings of the study are:

1. A confirmed infectious focus was identified in 2.6% of patients within seven days after the procedure, yet ABT was initiated in 11.4%.
2. Patients receiving ABT had more comorbidities, reduced functional capacity and more procedural complications. However, these parameters were not predictive of infection.
3. Despite its minimally invasive approach, distinct inflammatory reactions were observed post-TAVR, with BT and WBC peaking on day 1, and CRP levels on day 3.
4. Within the group of patients receiving ABT, the trends for BT, WBC, and CRP in the first three days after TAVR were similar regardless of the presence of an actual infection, underscoring the challenge of distinguishing between pronounced inflammation and true infection.
5. Nonetheless, combining these parameters within a risk stratification model (RIAT score) substantially enhances the predictability of actual infections, demonstrating its potential value as an additional component in clinical decision-making.

### Incidence of post-procedural infections and reasons for ABT administration

The reported incidence of in-hospital and early postoperative infections within 30 days after TAVR has decreased from up to 20% in the early 2000’s to about 11% more recently [4, 13, 15]. This decline might be attributed to increasing experience with TAVR and innovations in procedural techniques [23, 24]. In our study, the incidence of infectious complications after TAVR was only 2.6% within the first seven days. Although this limited observation period affects comparability with other studies, our findings are in line with general in-hospital infection rates (about 4%) in western countries [16, 17]. While TAVR is predominantly performed in older patients, who are more prone to infectious complications, this does not fully explain the discrepancies in incidence rates [25]. Interestingly, the frequency of ABT administration (11.4%) in our study mirrors the current reported infection rates after TAVR, suggesting substantial antibiotic overuse in this population. The implications of this overuse are significant: Adverse reactions range from allergic responses to severe disruptions in gut flora, including *Clostridium difficile* infections, which have 30-day mortality rates between 6 and 11% and 20–37% in an ICU setting [26, 27]. Furthermore, the indiscriminate use of ABT, particularly broad-spectrum types, contributes to the global issue of escalating antibiotic resistance [11]. In fact, ABT-resistant infections contributed to an estimated 4.95 million deaths globally in 2019 [9]. 

An important aspect of our study was understanding the factors leading to ABT initiation post-TAVR in addition to inflammatory parameters. Patients with ABT presented with a higher prevalence of comorbidities such as atrial fibrillation, COPD, and limited functional capacity, as indicated by a higher NYHA FC. Additionally, these patients had significantly more major procedural complications. This pattern indicates a possible tendency to a more liberal ABT use in patients with a perceived higher risk profile, as these factors have been consistently associated with adverse outcomes after TAVR [28–32]. However, our data do not support this approach, as these factors were not associated with early infections post-TAVR.

### Post-TAVR inflammatory reaction

A notable factor contributing to the liberal use of ABT may be the still limited understanding of the normal physiological responses following the procedure. Our study’s analysis provides crucial physiological insights. We noted distinct trends in BT, WBC, and CRP, with BT and WBC peaking on the first day post-procedure, and CRP on day 3. For patients with confirmed infectious foci (ABT_cf._), these markers showed higher values and delayed peaks as compared to the rest of the cohort, indicative of a prolonged inflammatory reaction beyond the typical postoperative response. A key observation was the similarity in the trajectories of BT, WBC, and CRP during the first three days post-TAVR between patients with confirmed infections (ABT_cf._) and those receiving ABT without retrospectively confirmed infections (ABT_nf_). This similarity poses a clinical challenge in distinguishing between pronounced postoperative inflammation and actual infection during this early phase, especially when considering each parameter independently.

From day 3 to 7 post-TAVR, a significant divergence in the trends of all three parameters was noted between those two groups. Patients in the ABT_cf._ cohort exhibited delayed declines in these markers, indicative of a sustained inflammatory state. This divergence identifies this later phase as critical for the reevaluation of ABT.

### Predictors of infection

Given that a significant proportion (73.1%) of ABT was initiated within the first three days post-TAVR, and the importance of rapidly differentiating pronounced inflammatory reactions from true infections during this early period, we identified predictors of infection by analyzing inflammatory markers during this critical period, along with clinically relevant parameters and comorbidities. Interestingly, neither baseline functional capacity nor comorbidities were associated with the occurrence of infections. Thus, the increasingly minimally invasive approach of TAVR, combined with only mild sedation, early mobilization, and shorter hospital stays, might mitigate the impact of functional capacity and comorbidities on the occurrence of infections in current TAVR recipients [18, 24, 33–35]. However, a rise in BT ≥ 0.2 °C, elevated WBC counts ≥ 12 × 10^9^ /L, and CRP levels ≥ 80 mg/L emerged as independent predictors of infectious complications post-TAVR.

### Integrated risk assessment

The similarity in trajectories of the identified predictors between individuals with pronounced inflammatory reaction and those with true infections limits the utility of interpreting these parameters independently. Furthermore, given the minimal impact of comorbidities and baseline characteristics on infection prediction and their limited additive discriminative power, we aimed to integrate the inflammatory reaction parameters into a comprehensive model. This led to the development of the RIAT score as an effective risk assessment tool.

In developing the RIAT score, we employed a graded combination of the three identified predictors, assigning different point values to each. This approach enabled the creation of a straightforward and user-friendly assessment tool. Upon its establishment in training and validation cohorts, the RIAT score demonstrated excellent performance across the overall patient cohort. Notably, with an increasing score from 0 to 8, there was a steep rise in the probability of infection. Thus, RIAT emerges as a pragmatic tool to efficiently estimate the likelihood of true infections and guide clinical decision-making regarding initiation of ABT.

### Limitations

The single center design of this study may limit its generalizability, warranting an external validation of the RIAT score. Retrospective analyses are inherently constrained by the accuracy and completeness of recorded data, particularly in identifying post-procedural infections. Another limitation is the focus on the early postoperative period, specifically within seven days post-TAVR, which may overlook late-onset infections, restricting the reflection of the entire spectrum of post-TAVR infectious complications. Strict diagnostic criteria for identifying infections may have led to the exclusion of some actual infections, potentially underestimating their true incidence. Patients receiving ABT or with identified infectious foci had longer hospital stays and more frequent blood testing, possibly biasing later measurements of inflammatory markers towards the end of the observation period.

## Conclusion

TAVR recipients exhibit distinct elevations in inflammatory parameters that should not be misinterpreted as infections without further clinical evidence. When clinical suspicion arises, the proposed RIAT score can support the integrative interpretation of inflammatory markers, aiding in the differentiation between inflammatory reactions and true infections. An increased score should prompt a thorough diagnostic workup to identify potential infectious foci and guide the initiation of targeted antibiotic therapy when appropriate. Once ABT has been initiated, the period from day 3 post-TAVR represents a critical time for its reevaluation and potential discontinuation, considering the clinical presentation, diagnostic results, and further trajectories of inflammatory markers. While the single-center design of this study may limit generalizability, the RIAT score’s robust internal validation provides a strong foundation for future external evaluation. Implementing this strategy will ensure rational antibiotic use, ultimately improving patient care in TAVR recipients and reducing antibiotic resistance.

## Electronic supplementary material

Below is the link to the electronic supplementary material.

Supplementary Material 1

## Data Availability

The data that support the findings of this study are not openly available due to reasons of sensitivity and are available from the corresponding author upon reasonable request. Data are located in controlled access data storage at University Hospital Cologne.
