# Publisher Correction: Evaluation of systemic inflammatory response following transcatheter aortic valve replacement: a pathway to rational antibiotic use

**DOI:** 10.1007/s15010-025-02511-1

**Published:** 2025-03-19

**Authors:** Henning Guthoff, Valerie Lohner, Ute Mons, Julia Götz, Hendrik Wienemann, Jan Wrobel, Stephan Nienaber, Sascha Macherey-Meyer, Philipp von Stein, Stephan Baldus, Matti Adam, Maria Isabel Körber, Norma Jung, Victor Mauri

**Affiliations:** 1 Heart Center, Department III of Internal Medicine, Faculty of Medicine and University Hospital Cologne, University of Cologne, Kerpener Str. 62, 50937 Cologne, Germany; 2 Cardiovascular Epidemiology of Aging, Department III of Internal Medicine, Faculty of Medicine and University Hospital Cologne, University of Cologne, Cologne, Germany; 3 Division of Infectious Diseases, Department I of Internal Medicine, Faculty of Medicine and University Hospital Cologne, University of Cologne, Cologne, Germany

**Correction: Infection (2025)**

10.1007/s15010-025-02485-0

Due to a typesetting error In this article the following note is missing:

Norma Jung and Victor Mauri contributed equally

The Original Article has been corrected.

