# Supplementary material for: Evaluation of systemic inflammatory response following transcatheter aortic valve replacement: a pathway to rational antibiotic use
Source: Infection. 2025 Feb 7;53(5):1725–35. doi: 10.1007/s15010-025-02485-0 (PMC12460358; doi:10.1007/s15010-025-02485-0)
Supplement: Supplementary file 1 — Supplementary Material 1 [file 15010_2025_2485_MOESM1_ESM.docx]

**Supplemental Material**

**Evaluation of Systemic Inflammatory Response following Transcatheter Aortic Valve Replacement: A Pathway to Rational Antibiotic Use**

Henning Guthoff MD^1^, Valerie Lohner PhD^2^, Ute Mons PhD^2^, Julia Götz MD^3^, Hendrik Wienemann MD^1^, Jan Wrobel MD^1^, Stephan Nienaber MD^1^, Sascha Macherey-Meyer MD^1^, Philipp von Stein MD^1^, Stephan Baldus MD^1^, Matti Adam MD^1^, Maria Isabel Körber MD^1^, Norma Jung MD*^3^, and Victor Mauri MD*^1^

*contributed equally

Corresponding author:

Victor Mauri, MD; victor.mauri@uk-koeln.de

Heart Center, University Hospital Cologne, Kerpener Str. 62, 50937 Cologne, Germany

^1^ Department III of Internal Medicine, Faculty of Medicine and University Hospital Cologne, University of Cologne, Cologne, Germany.

^2^ Cardiovascular Epidemiology of Aging, Department III of Internal Medicine, Faculty of Medicine and University Hospital Cologne, University of Cologne, Cologne, Germany.

^3^ Division of Infectious Diseases, Department I of Internal Medicine, Faculty of Medicine and University Hospital Cologne, University of Cologne, Cologne, Germany.

| **Condition** | **Criteria** | **Reference** |
| --- | --- | --- |
| Urinary Tract Infection | Positive urine culture (≥10^5^ CFU/ml; ≥10^3^ CFU/ml if symptoms are present)  Symptoms include dysuria, frequent urination, urgency, or suprapubic pain | Adapted from German Society of Urology S3 Guideline^1^ |
| Cutaneous Bacterial Infection | Local redness, swelling, and warmth in the absence of an alternative diagnosis (e.g., eczema, stasis dermatitis)  In case of open wounds: pus or secretion discharge and/or positive swab culture | Adapted from IDSA Guidelines^2^ |
| Bloodstream Infection | Positive blood cultures indicative of a bloodstream infection with respective signs and symptoms of infection (e.g., fever, chills, or hypotension) | Adapted from Sepsis-3 Definitions^3^ |
| Pneumonia | One major (newly developed infiltrate in chest X-ray) and two minor criteria: - Fever (≥38°C) or hypothermia (≤36°C) - Leukocytosis (WBC ≥11x10⁹/L) or leukopenia (WBC <4x10⁹/L) - Purulent sputum - Physical signs suggestive of infiltration (e.g., bronchophony, or vocal fremitus) - Pathogen detection (blood culture, sputum, bronchial secretions, or pleural fluid) | Adapted from ATS/IDSA Guidelines^4^ |
| Encephalitis | One major (altered mental status lasting ≥24 hours with no alternative cause) and two minor criteria: - Fever ≥38°C within 72 hours before or after presentation - New seizures - New focal neurologic findings - CSF with leukocyte count ≥5 cells/mm³. - Imaging findings (MRI/CT) showing signs of new brain inflammation - EEG abnormalities consistent with encephalitis and no alternative explanation | Adapted from IDSA Guidelines^5^ |
| Spontaneous Bacterial Peritonitis | >250 Granulocytes/μL in ascites sample | Adapted from EASL Guidelines^6^ |

**Table S1: Diagnostic criteria used for the retrospective identification of infectious diagnoses in the study**

Abbreviations: ATS – American Thoracic Society, CFU – Colony-Forming Units, CSF – Cerebrospinal Fluid, CT – Computed Tomography, EEG – Electroencephalogram, EASL – European Association for the Study of the Liver, IDSA – Infectious Diseases Society of America, MRI – Magnetic Resonance Imaging, WBC – White Blood Cells

References for Table S1:

1. German Society of Urology (DGU). S3 Guideline: Epidemiology, Diagnosis, Therapy, Prevention, and Management of Uncomplicated, Bacterial, Community-Acquired Urinary Tract Infections in Adults – Update 2024.

2. Stevens DL, Bisno AL, Chambers HF, et al. Practice Guidelines for the Diagnosis and Management of Skin and Soft Tissue Infections: 2014 Update by the Infectious Diseases Society of America. Clin Infect Dis. 2014;59(2):e10–e52.

3. Singer M, Deutschman CS, Seymour CW, et al. The Third International Consensus Definitions for Sepsis and Septic Shock (Sepsis-3). JAMA. 2016;315(8):801–810.

4. Metlay JP, Waterer GW, Long AC, et al. Diagnosis and Treatment of Adults with Community-acquired Pneumonia. Am J Respir Crit Care Med. 2019;200(7):e45–e67.

5. Tunkel AR, Glaser CA, Bloch KC, et al. The Management of Encephalitis: Clinical Practice Guidelines by the Infectious Diseases Society of America. Clin Infect Dis. 2008;47(3):303–327.

6. European Association for the Study of the Liver. EASL Clinical Practice Guidelines for the management of patients with decompensated cirrhosis. J Hepatol. 2018;69(2):406–460.

|  |  |  |  |
| --- | --- | --- | --- |

| Antibiotics used | Number of patients |
| --- | --- |
| *Amoxicillin/Clavulanic acid* | 6 |
| *Ampicillin* | 1 |
| *Ampicillin/Sulbactam* | 16 |
| *Cefazolin* | 1 |
| *Cefpodoxime* | 4 |
| *Ceftazidime* | 1 |
| *Ceftriaxone* | 16 |
| *Cefuroxime* | 5 |
| *Ciprofloxacin* | 11 |
| *Clarithromycin* | 3 |
| *Co-trimoxazole* | 6 |
| *Flucloxacillin* | 3 |
| *Fosfomycin* | 3 |
| *Gentamicin* | 1 |
| *Meropenem* | 11 |
| *Moxifloxacin* | 3 |
| *Nitrofurantoin* | 1 |
| *Piperacillin/Tazobactam* | 96 |
| *Rifampicin* | 2 |
| *Vancomycin* | 7 |

**Table S2: List of antibiotics used in the study cohort, along with the respective number of patients who received each antibiotic.**

| *Diagnosis* | Initial working diagnosis (n) | Retrospectively  confirmed diagnosis (n) |
| --- | --- | --- |
| *Infection of unknown origin* | 59 | 0 |
| *Urinary tract infection* | 30 | 14 |
| *Cutaneous infection* | 5 | 3 |
| *Bloodstream infection* | 4 | 5 |
| *Pneumonia* | 45 | 10 |
| *Encephalitis* | 1 | 0 |
| *Spontaneous Bacterial Peritonitis* | 1 | 1 |

**Table S3**: **List of infectious diagnoses along with the respective number of patients, separated by initial working diagnosis and retrospectively confirmed diagnosis.**

**Figure S1: Number of patients within the ABT_cf_ group categorized as no sepsis, sepsis, or septic shock according to Sepsis-3 criteria. The red portion indicates deceased patients.**

**Figure S2: Proportion of appropriate diagnostic testing for each suspected infection category.**

| Patient A |
| --- |
| *Staphylococcus aureus (MSSA)* |
|  |
| Patient B |
| *Staphylococcus aureus (MSSA)* |
|  |
| Patient C |
| *Enterococcus faecalis* |
|  |
| Patient D |
| *Klebsiella pneumoniae* |
|  |
| Patient E |
| *Staphylococcus aureus (MSSA)* |

**Table S4: List of pathogens detected in blood cultures from patients diagnosed with bloodstream infections.**

Abbreviations: MSSA – Methicillin-Sensitive *Staphylococcus aureus*.

| *Parameter* | Training  cohort | Validation cohort | *P* |
| --- | --- | --- | --- |
| *N* | 879 | 396 | 0.806 |
| *Age - years* | 81.5±6.2 | 81.6±5.8 | 0.107 |
| *Females* | 406 (46.2%) | 163 (41.1%) | 0.378 |
| *BMI - kg/m^2^* | 27.1±5.3 | 26.8±4.8 | 0.284 |
| *Average BT  over 7d post-TAVI - °C* | 36.7±0.4 | 36.7±0.4 | 0.225 |
| *Maximum BT*  *within 7d post-TAVI - °C* | 37.4±0.6 | 37.4±0.6 | 0.173 |
| *Average WBC*  *over 7d post-TAVI - x10^9^/L* | 8.8±4.7 | 8.5±2.5 | 0.559 |
| *Maximum WBC*  *within 7d post-TAVI - x10^9^/L* | 10.7±6.2 | 10.5±4.7 | 0.898 |
| *Average CRP level over 7d post-TAVI - mg/L* | 30.6±30.6 | 30.4±29.5 | 0.905 |
| *Maximum CRP level within 7d post-TAVI - mg/L* | 55.3±54.6 | 55.0±52.0 | 0.921 |
| *Fever post-TAVI (BT≥38°C)* | 143 (16.4%) | 67 (17.1%) | 0.336 |
| *GFR - ml/min* | 59±21 | 60±20 | 0.806 |
| *NYHA FC* |  |  | 0.601 |
| *I* | 24 (2.8%) | 14 (3.7%) |  |
| *II* | 233 (27.5%) | 98 (25.9%) |  |
| *III* | 531 (62.7%) | 245 (64.8%) |  |
| *IV* | 59 (7.0%) | 21 (5.6%) |  |
| *EuroSCORE II - %* | 4.3±4.0 | 4.2±3.8 | 0.677 |
| *STS score - %* | 4.5±4.5 | 4.0±3.4 | 0.051 |
| *Comorbidities* |  |  |  |
| *Hypertension* | 769 (87.8%) | 337 (85.8%) | 0.362 |
| *Diabetes mellitus* | 247 (28.2%) | 106 (27.0%) | 0.702 |
| *Peripheral arterial disease* | 149 (17.0%) | 74 (18.8%) | 0.479 |
| *Coronary heart disease* | 528 (60.3%) | 237 (60.3%) | 1.000 |
| *Atrial fibrillation* | 345 (39.4%) | 160 (40.7%) | 0.700 |
| *COPD* | 100 (11.4%) | 39 (9.9%) | 0.490 |
| *Median length of hospital stay  - days* | 7 (5-10) | 7 (5-10) | 0.988 |
| *Median length of ICU stay  - days* | 2 (1-3) | 2 (1-3) | 0.527 |
| *Self-expanding device* | 576 (65.5%) | 264 (66.7%) | 0.139 |
| *Major complication* | 170 (19.3%) | 96 (24.2%) | 0.055 |

**Table S5: Baseline characteristics of the training and validation cohort for the Risk of Infection after TAVI (RIAT) score.** Major complications include new pacemaker implantation, stroke/TIA, and events classified as ≥ type 2 bleeding or major vascular complications as defined by VARC3.

Abbreviations: BMI – Body Mass Index, BT – Body Temperature, COPD – Chronic Obstructive Pulmonary Disease, CRP – C-Reactive Protein, GFR – Glomerular Filtration Rate (according to Chronic Kidney Disease Epidemiology Collaboration), ICU – Intensive Care Unit, NYHA FC – New York Heart Association Functional Class, TIA – Transient Ischemic Attack, VARC3 – Valve Academic Research Consortium-3, WBC – White Blood Cell Count.

**Figure S3: ROC analysis and AUC calculation for the RIAT Score in identifying patients with an infectious focus across the specified patient cohorts.**

Abbreviations: AUC – Area Under the Curve, ROC – Receiver Operating Characteristics, RIAT – Risk of Infection After TAVI

| **RIAT Score** | **Sensitivity (%)** | **Specificity (%)** | **PPV (%)** | **NPV (%)** |
| --- | --- | --- | --- | --- |
| ≥ 4 | 45.4 | 92.4 | 13.7 | 98.5 |
| ≥ 7 | 18.2 | 99.6 | 54.5 | 97.9 |

# Table S6: Sensitivity, specificity, positive predictive value (PPV), and negative predictive value (NPV) for the given cutoff values of the RIAT Score.
